# Mouse Models of *C9orf72* Hexanucleotide Repeat Expansion in Amyotrophic Lateral Sclerosis/ Frontotemporal Dementia

**DOI:** 10.3389/fncel.2017.00196

**Published:** 2017-07-06

**Authors:** Ranjan Batra, Chris W. Lee

**Affiliations:** ^1^Department of Cellular and Molecular Medicine, University of California, San Diego, La JollaCA, United States; ^2^Institute for Genomic Medicine, University of California, San Diego, La JollaCA, United States; ^3^Atlantic Health System, MorristownNJ, United States; ^4^Biomedical Research Institute of New Jersey, Cedar KnollsNJ, United States

**Keywords:** C9orf72, frontotemporal dementia, amyotrophic lateral sclerosis, mouse model, RNA toxicity, adeno-associated viral vector, bacterial artificial chromosome, microsatellite repeat expansion

## Abstract

The presence of hexanucleotide repeat expansion (HRE) in the first intron of the human *C9orf72* gene is the most common genetic cause underlying both familial amyotrophic lateral sclerosis (ALS) and frontotemporal dementia (FTD). Studies aimed at elucidating the pathogenic mechanisms associated of *C9orf72* FTD and ALS (C9FTD/ALS) have focused on the hypothesis of RNA and protein toxic gain-of-function models, including formation of nuclear RNA foci containing GGGGCC (G_4_C_2_) HRE, inclusions containing dipeptide repeat proteins through a non-canonical repeat associated non-ATG (RAN) translation mechanism, and on loss-of-function of the C9orf72 protein. Immense effort to elucidate these mechanisms has been put forth and toxic gain-of-function models have especially gained attention. Various mouse models that recapitulate distinct disease-related pathological, functional, and behavioral phenotypes have been generated and characterized. Although these models express the *C9orf72* HRE mutation, there are numerous differences among them, including the transgenesis approach to introduce G_4_C_2_-repeat DNA, genomic coverage of *C9orf72* features in the transgene, G_4_C_2_-repeat length after genomic stabilization, spatiotemporal expression profiles of RNA foci and RAN protein aggregates, neuropathological features, and neurodegeneration-related clinical symptoms. This review aims to (1) provide an overview of the key characteristics; (2) provide insights into potential pathological factors contributing to neurotoxicity and clinical phenotypes through systematic comparison of these models.

## *C9orf72* Hexanucleotide Repeat Expansion in FTD and ALS

Frontotemporal Dementia (FTD) is a devastating neurodegenerative disorder with heterogeneous clinical, genetic, and pathological features. FTD patients develop clinical symptoms like changes in behavior, personality, or language due to the progressive degeneration of the frontal and anterior temporal cortices (Snowden et al., 2007). The development of clinical phenotypes correlates with brain regions undergoing neurodegeneration (Sieben et al., 2012). These include behavioral-variant FTD (bvFTD), and two language variants including semantic variant primary progressive aphasia (svPPA), and non-fluent/agrammatic variant primary progressive aphasia (nfvPPA). The prevalence of FTD is approximately 15 to 22 cases per 100,000 people. The age of onset is 45 to 65 years with a range between 21 and 85 years of age, and disease duration of 8 years (range 2–20) after onset of symptoms (Hodges et al., 2003; Johnson et al., 2005; Neary et al., 2005; Garcin et al., 2009).

Amyotrophic lateral sclerosis (ALS), also known as Lou Gehrig’s disease, is a motor neuron disease (MND). It is a fatal neurodegenerative disorder characterized by prominent degeneration of upper and lower motor neurons (MNs) in the primary motor cortex and the anterior horn of the spinal cord, respectively. ALS patients develop muscle weakness, spasticity, and atrophy resulting in paralysis, often leading to fatality through respiratory failure (Haverkamp et al., 1995; Mitchell and Borasio, 2007). Age of onset is between 50 and 60 years of age with a disease duration of 2–5 years from the onset of symptoms (Kiernan et al., 2011). The prevalence of ALS is 4.7 cases per 100,000 people per year worldwide (Beghi et al., 2006) with about 60% higher incidence rate in males (Mehta et al., 2016).

GGGGCC (G4C2) hexanucleotide repeat expansion (HRE) mutations in Chromosome 9 open reading frame 72 gene (*C9orf72*) are the most common genetic cause of FTD and ALS (abbreviated as C9FTD/ALS thereafter) (DeJesus-Hernandez et al., 2011; Renton et al., 2011). G4C2 HREs account for approximately 26 and 34% of familial FTD and ALS cases, respectively, in the United States and ∼6% sporadic FTD and ALS cases worldwide (van Blitterswijk et al., 2013). The high prevalence of this mutation causing both sporadic and familial forms of ALS and FTD is highly intriguing and has led to a significant interest in pursuing pathogenic mechanisms associated with the G4C2-repeat expansion. While the affected mutation carriers can have up to 5000 repeats in the frontal cortex, non-mutation carriers have less than 23 repeats (DeJesus-Hernandez et al., 2011). Interestingly, a high variability in repeat size is present between different brain region and tissues (van Blitterswijk et al., 2013). However, a correlation between repeat length and severity has not been consistently detected among studies (van Blitterswijk et al., 2013; Dols-Icardo et al., 2014; Nordin et al., 2015). The G4C2-repeat expansions occur in the first intron between two alternatively spliced non-coding exons, exons 1a and 1b. A series of alternative splicing events produce 3 known pre-mRNA transcripts - variants 1 (NM_145005) and 3 (NM_001256054) contain exon 1a and intronic G4C2-repeat expansion; whereas variant 2 (NM_018325) contains exon 1b and G4C2-repeat expansion in the promoter region. Variants 2 and 3 encode a longer isoform of the C9orf72 protein (481 amino acids) that includes exons 2 to 11. In contrast, variant 1 encodes a shorter isoform (222 amino acids) with exons 2 to 5.

The goal of this review is to: (1) provide an up-to-date summary of the key findings of expanded G4C2 HRE mouse models that potentially recapitulate human C9FTD/ALS molecular pathology and phenotypes; (2) provide insights into potential pathological factors contributing to neurotoxicity and clinical phenotypes of C9FTD/ALS through careful comparison of these models (**Table 1**).

**Table 1:** Summary table of key features in the published *C9orf72* HRE mouse models.

	*C9ORF72*Mouse Models	AAV-G4C2-66AAV-G4C2-2(Chew et al., 2015)	BAC-C9-500/300(Peters et al., 2015)		BAC-C9-(100–1000) F112BAC-C9-(100–1000) F113BAC-C9-15(O’Rourke et al., 2015)		BAC-C9-450 ABAC-C9-450 BBAC-C9-450 CBAC-C9-110(Jiang et al., 2016)		BAC-C9-500BAC-C9-500/32BAC-C9-36/29BAC-C9-37(Liu et al., 2016)	
**A**	**Strain**	**C57BL/6J**	**SJL/BL6**		**C57BL/6J**		**C57BL6/C3H**		**FVB/NJ**	
**B**	**Transgene DNA applied to generate the mouse models**	AAV2/9 vector with CBA promoter driving transcription of (G4C2) 2 or (G4C2) 66 with 119 bp 5′ flanking region and 100 bp 3′ flanking region of human *C9ORF72* gene	BAC DNA containing partial coding region of human *C9ORF72* gene (exon 1 to 6) including a (G4C2) 500 region and 141 Kb of 5′ upstream region		BAC DNA containing complete coding region of human *C9ORF72* gene (exon 1 to 11) including a (G4C2) 800 region, a 110 Kb 5′ upstream region and a 20 Kb 3′ downstream region		BAC DNA containing partial coding region of human *C9ORF72* gene (exon 1 to 5) including a (G4C2) 450 region and 140 Kb of 5′ upstream region		BAC DNA containing complete coding region of human *C9ORF72* gene (exon 1 to 11) including a (G4C2) 830+ region, a 52 Kb upstream region and a 19 Kb downstream region	
**C**	**# of mouse line and G4C2-repeat size**	1. 66 repeats2. 2 repeats (control)	1. Mix of 500/300 repeats2. Non-transgenic control		1. Mix of 100 to 1000 repeats (F112)2. Mix of 100 to 1000 repeats (F113)3. 15 repeats (control)		1. 450 repeats (line A)2. 450 repeats (line B)3. 450 repeats (line C)4. 110 repeats5. Non-transgenic control		1. 500 repeats2. 500/32 repeats3. 36/29 repeat4. 37 repeats (control)	
**D**	**Transgene transcript levels in the mice (compare to levels of endogenous mouse *C9ORF72* RNA)**		1× (similar levels); also similar to *C9ORF72* RNA levels in human FCx		Not determined; Assume to have similar levels to endogenous *C9ORF72* RNA levels based on total *C9ORF72* protein levels in cortex detected by western blot		**Het**450A: 1×450B: 3×450C: 4.5×110: 2.5×	**Homo**450C: 8–9×	**Sense (vs. non-TG)**500: 1×500/32: 2×36/29: 3×37: 0.5×	**Antisense (vs. C9-37)**500: 40×500/32: 22×36/29: 5×37×: 1×
**E**	**Sense RNA foci (G4C2-repeat)**	In 40–54% cells in cortex, MCx, Hp-CA, Th and Cb Purkinje layer. Less in Hp-DG, Cb granular layer and SC of AAV-G4C2-66 mice. Non-detectable in AAV-G4C2-2 mice. (**6 month’s old**)	Sense foci was detected abundantly in brain and SC in the BAC-C9-500/300 mice at **3, 10, and 24 months of age**. About 50 and 80% cells with sense foci in MCx internal pyramida layer and SC motor neurons, respectively, **at 10 months** of age.		In 40–60% cells in Purkinje layer of Cb, Hp-DG, FCx and PMCx of BAC-C9-(100–1000) mice (Line F112, **3 months**).No significant change of distribution and % of foci +ve cells from **3–6 month’s old**.		Sense foci were abundant in FCx, Hp-DG, RSCx and Cb in BAC-C9-450B mice at **2 months**; 52% and 81% of cells in Hp-DG of **6 month’s old** heterozygous and homozygous BAC-C9-450C mice, respectively. Non-detectable in BAC-C9-110 mice.		In nearly all NeuN positive cells of cortex, Hp, Cb and SC in acute-progress mice. Non-detectable in BAC-C9-36/29 and BAC-C9-37 mice. (**2 month’s old**)	
**F**	**Antisense RNA foci (G2C4-repeat)**		Similar distribution but much less antisense foci were detected compared to sense RNA foci in **10 and 24 month’s old** BAC-C9-500/300 mice.		In 40–80% cells in Purkinje layer of Cb, Hp-DG, FCx and PMCx in C9-100–1000 mice (Line F112, **3 months**).No significant change of distribution and % cells with RNA foci from **3 months to 6 months of age**.		Antisense foci were detected in FCx, Hp, Cb and SC in C9-450B mice at **2 months**; 29% and 42% of cells in Hp-DG of **6 month’s old** heterozygous and homozygous BAC-C9-450C mice, respectively. Non-detectable in the BAC-C9-110 mice.		Abundant levels only found in degenerated regions, including MCx, Hp-CA, Hp-DG, Cb Purkinje cells and interneurons in lateral and posterior horns of SC, except lower motor neurons in SC in **2 month’s old** acute-progress mice.	
**G**	**Soluble poly(GP) levels in CNS (ng/mg protein)**	Whole brain: 17,000 ng/mg protein (6 month’s old AAV-G4C2-66 mice)	**4 months**Cortex: 50Hp: 50Cb: 100SC: 30Mb: 50Hb: 30	**24 months**Cortex: 20Hp: 20Cb: 10SC: <10Mb: 15Hb: <10	**F112**Cortex: 300–400Other regions:notdetermined(**6 month’s old**)	**F113**Cortex: 200Hp: 100Cb: 400SC: 100Human C9FCx: 250–400(**6 month’s old**)	**Het** **C9-450C mice**Cortex: 45Cb: 10SC: 2(**6 month’s old**)	**Homo C9-450C mice**Cortex: 120Cb: 30SC: 20(**6 month’s old**)		
		Non-detectable in the AAV-G4C2-2 mice								
					Non-detectable in the **BAC-C9-15 mice**		Non-detectable in the **BAC-C9-110 mice**			
**H**	**C9RAN protein pathology**	High to moderate levels of poly(GA) and poly(GP) aggregates, low levels of poly(GR) aggregates were found in cortex, Hp-CA and thalamus regions. Low levels of poly(GA) and poly(GP) aggregates were found in Cb Purkinje cells and SC. (**6 month’s old** AAV-G4C2-66 mice)	Poly(GP) aggregates were found throughout the brain including cortex, striatum and Cb in **10 month’s old** BAC-C9-500/300 mice. Poly(GP) aggregates were more frequently found and apparently larger size at **24 month’s old** compared to **10 month’s old.**		Poly(GP) aggregates were “rarely” found in **6 month’s old** BAC-C9-(100–1000) mice. But insoluble poly(GP) in cortex homogenate could be detected by immunoassay at **6 months**. More frequently in cortex, Hp-DG and Cb of **20 month’s old** BAC-C9-(100–1000) mice.		Poly(GA), poy(GP) and poly(GR) aggregates were detected in RSCx as early as **3 month of age.** Size and % +ve cells of poly(GA) aggregate increased 2× and 10×, respectively, **from 6 to 22 months of age**, in heterozygous BAC-C9-450C mice. Abundant poly(GP) and poly(GA) aggregates found in RCx > Hp-DG > FCx in **22 month’s old** mice (Up to 30% cells in RCx). Homozygous had five times more poly(GA) aggregates than heterozygous BAC-C9-450C mice in RCx at **3 and 6 months of age**.		Abundant poly(GA) aggregates were detected throughout brain (including Hp-DG, Cb Purikinje cells, motor cortex and BS) and SC in all BAC-C9 lines except BAC-C9-37 line in end-stage acute-progress mice (**∼5–7 months**) but rarely found in age-matched asymptomatic mice from same line. Poly(GP) aggregates also detected in cortex, Th and SC interneurons of end-stage acute-progress mice. Size and number of poly(GA) aggregates in RSCx were positively correlated with symptoms severity in **5 month’s old** acute-progress BAC-C9-500 mice.	
**I**	**TDP-43 pathology**	Nuclear and less frequent cytoplasmic pTDP-43 aggregates were found in about 7–8% of cells in cortex, motor cortex and Hp of AAV-G4C2-66 mice. Western blot confirmed	Non-detectable		Non-detectable		Increased phosphorylated TDP-43 levels in SDS insoluble fraction of cortex from **22 month’s old** heterozygous BAC-C9-450C mice.		Cytoplasmic and nuclear TDP-43 aggregates were found in degenerating neurons (including Hp-CA1 and MCx) of end-stage acute-progress mice (**5–7 months**) but not in age-matched asymptomatic mice from same line.	
		pTDP-43 was in SDS-soluble monomeric and dimeric forms. (**6 month’s old**)								
**J**	**Neuronal loss**	About 17 and 23% loss of NeuN positive neurons in whole cortex and motor cortex, respectively. About 11% loss of Cb Purkinje cells. **(6 month’s old** AAV-G4C2-66 mice)	Non-detectable		Non-detectable		About 10% neuronal loss in Hp-DG and Hp-CA1 regions of **12 month’s old** heterozygous BAC-C9-450B and BAC-C9-450C mice.		**Acute-progress (5–7 months)** Extensive neuronal loss throughout layer II/ II of cortex; Significant to complete loss of CA and DG regions; Loss of Cb Purkinje cells (65%); Interneurons loss in SC posterior horn (∼33%)	
									**Slow-progress (12–17 months)** Mild focal degeneration in neocortex; No neuronal loss in Hp; Loss of Cb Purkinje cells (∼20%)	
**K**	**Motor unit injuries**	Upper motor neurons loss in **6 month’s old** AAV-G4C2-66 mice. No SC motor neurons loss was detected.	Non-detectable; Analyzed by motor unit number estimations, motor units size or compound motor action potential in **24 month’s old** BAC-C9-500/300 mice.		Non-detectable		Non-detectable		**Acute-progress (5–7 months)** Upper motor neurons loss in layer II/III (75%) and layer V (57%) of MCx; Lower motor neurons loss in LSC (∼50%); Denervation NMJ in TA (66%) and diaphragm (49%) muscles	
									**Slow-progress (12–17 months)** Lower motor neurons loss in LSC (30%); Subtle NMJ and SC motor axons abnormalities e.g. axonal swelling, reduced fiber sizes etc.	
**L**	**Behavioral phenotypes**	Increased anxiety; Sociability deficit; Reduced motor function; About 10% body weight reduction in female AAV-G4C2-66 mice. (**6 month’s old**)	Non-detectable: C9-500/300 mice were analyzed by behavioral tests for motor function (**3–24 months**) and sociability (**18–22 months**).		Non-detectable; F112 mice were analyzed by behavioral tests for motor function, anxiety, sociability, memory and novelty-seeking behavior at **18 months.**		Spatial learning deficit and increased anxiety in heterozygous BAC-C9-450B and BAC-C9-450C mice. (**12 month’s old**)		**Acute-progress (4–7 months)** Weigh loss; Motor function deficit including gait abnormality and reduced grip strength; Hindlimb paralysis and early death	
									**Slow-progress (12–17 months)** Kyphosis, reduced	
									activity, clasping, intermittent seizures, and increased anxiety	
**M**	**Others**	Activated microgliosis and increased astrogliosis, p62 and ubiquitinated inclusions found in **6 month’s old** AAV-G4C2-66 mice.	No significant change of transcriptome gene expression profile by sequencing total RNA from FCx of **6 month’s old** BAC-C9-500/300 mice.		1. Detection of nucleolar stress;2. Alternation in transcriptome gene expression profile;3. Did not detect sequestration of known G4C2-repeat binding RBPs by RNA foci in the BAC-C9-(100–1000) mice.				1. Activated microglia and astrocytosis in degenerated brain regions of end-stage acute-progress mice.2. Sexual difference: female C9 mice with more early-onset and severe phenotypes including hindlimb paralysis and early death between**5–7 months**.	

*Het, heterozygous; Homo, homozygous; TG, transgenic; FCx, frontal cortex; MCx, motor cortex; PFCx, prefrontal cortex; Hp, hippocampus; DG, dentate gyrus; CA, *cornu ammonis*; RSCx, retrosplenial cortex; Th, thalamus; Cb, cerebellum; SC, spinal cord; LSC, lumbar spinal cord; Mb, midbrain; Hb, hindbrain; NMJ, neuromuscular junction; TA, tibialis anterior*.

## Potential Disease Mechanisms

Despite prolific research efforts since the discovery of the *C9orf72* G4C2 HRE mutation, the exact pathogenic mechanisms remain elusive. Three major mechanisms have been suggested: (1) Toxicity mediated by bidirectional transcription of RNA containing G4C2 HRE is the most prominent mechanism and thought to play an important role. HRE containing transcripts form nuclear RNA foci that may sequester RNA-binding proteins (RBPs) (Harley et al., 1992; Almeida et al., 2013; Donnelly et al., 2013; Lee et al., 2013; Mori et al., 2013b; Reddy et al., 2013; Sareen et al., 2013; Xu et al., 2013; Cooper-Knock et al., 2014) and (2) are susceptible to repeat-associated non-ATG (RAN) translation resulting in production of C9RAN dipeptide-repeat proteins, including poly(GA), poly(GR), and poly(GP) proteins from the sense transcripts, poly(GP), poly(PA), and poly(PR) from the antisense transcripts (Ash et al., 2013; Gendron et al., 2013; Mori et al., 2013a; Zu et al., 2013). Although RBP sequestration has been suggested by the aforementioned studies similar to the disease mechanisms of myotonic dystrophy (DM types I and II), no RBP loss-of-function mouse models that recapitulate the C9FTD/ALS phenotypes exist. Genome-wide RNA processing alterations are often observed downstream of RBP dysfunction (Charizanis et al., 2012; Batra et al., 2014). Indeed, widespread changes in alternative splicing and polyadenylation occur in C9ALS frontal cortex and cerebellum (Prudencio et al., 2015), suggesting an RBP dysfunction mechanism. (3) The third potential mechanism is *C9orf72* haploinsufficiency due to the inhibition of coding region translation by the *C9orf72* HRE. Nestin-Cre mouse model with neural-specific knockout of *C9orf72* show reduction in body weight but no neurotoxicity (Koppers et al., 2015). In addition, *C9orf72* knockout mouse models also demonstrated splenomegaly and lymphadenopathy phenotypes indicative of immune system dysregulation (Atanasio et al., 2016; Jiang et al., 2016; O’Rourke et al., 2016). At subcellular levels, accumulation of enlarged lysosomes was detected in *C9orf72* knockout mice (O’Rourke et al., 2016; Ji et al., 2017). Impaired autophagy functions and autophagy signaling pathways have also been identified in *C9orf72* knockout cell and mouse models (Amick et al., 2016; Ugolino et al., 2016; Yang et al., 2016; Ji et al., 2017). Given the emerging role of C9orf72 in autophagy and immune system functions, C9orf72 protein haploinsufficiency may implicate in disease processes through dysregulation of immune cell responses in central nervous system (CNS). Similar to other neurodegenerative disorders, the C9FTD/ALS can be potentially caused by the interplay of all the three mechanisms mentioned above.

Generation of mouse models expressing the G4C2-repeat expansion that recapitulate pathological and clinical features are crucial for elucidating disease mechanisms associated with C9FTD/ALS. These models will also provide *in vivo* systems to develop and evaluate biomarkers and therapeutics in preclinical studies.

## Aav-Mediated G4C2-Repeat Expression Mouse Model

Chew et al. (2015) published the first G4C2-repeat expansion mouse model in June 2015. In this model, transgene delivery was mediated by somatic transduction of adeno-associated virus (AAV) carrying the G4C2-repeat DNA to CNS in the C57BL/6J mouse genome. Transcription of a (G4C2)_66_ or a (G4C2)_2_ DNA with a 119 base-pair (bp) of the upstream 5′ region and 100 bp of the downstream 3′ region of the human *C9orf72* gene was driven by a chicken β-actin (CBA) promoter in an AAV2/9 vector. Bilateral intracerebroventricular (ICV) administration of AAV2/9-(G4C2)_66_ or the AAV2/9-(G4C2)_2_ was performed at post-natal day 0 to achieve widespread brain transduction. The fact that the G4C2-repeat transgene and the upstream flanking regions did not contain an ATG start codon allowed for the modeling of RAN translation of the (G4C2)_66_ transcripts similar to the C9FTD/ALS patients. Mice were tested for behavioral, pathological, and biochemical abnormalities at 6 months.

Sense G4C2-repeat transcripts were analyzed by RNA fluorescence *in situ* hybridization (FISH) and RNA foci were detected in about 50% of cell populations in cortex, motor cortex, hippocampus and cerebellar Purkinje layer of AAV-G4C2-66 mice but not in the AAV-G4C2-2 control mice. Fewer cells containing RNA foci were detected in the ventral horn of spinal cord, granular and molecular layers of cerebellum. Unfortunately, the antisense G2C4-repeat RNA foci were not analyzed in the study.

Consistent with the expression of the sense G4C2-repeat transcript, RAN-mediated translation of poly(GA), poly(GP), and poly(GR) peptides were detected in cortex, hippocampus and Purkinje cells by immunohistochemistry (IHC) staining. The spinal cord showed lesser loads of RAN-peptides consistent with having lesser RNA foci in that region. Poly(GA) mainly appeared as globular inclusions. Although discrete poly(GP) inclusions and poly(GR) inclusions could be detected occasionally, poly(GP) and poly(GR) staining frequently appeared as diffuse nuclear and diffuse cytoplasmic, respectively. Greater number of cells were stained positive for poly(GA) or poly(GP) than for poly(GR). Using a Meso-scale Discovery (MSD) platform-based immunoassay to measure soluble poly(GP) levels in whole brain homogenate of the AAV-G4C2-66 mice (Gendron et al., 2013), an ultra-high concentration of ∼17,000 ng/mg protein was detected, illustrating a robust production and translation of the (G4C2)_66_ transcripts.

Phosphorylated inclusions of the protein product of the RNA binding protein (RBP) gene *TARDBP*, are the most common neuropathological hallmark in ALS including C9ALS (Neumann et al., 2006). Interestingly, nuclear phosphorylated TDP-43 (pTDP-43) inclusions were found in approximately 7–8% of cells in cortex and hippocampus of the AAV-G4C2-66 mice but not in the AAV-G4C2-2 mice. Majority of the cells containing the pTDP-43 inclusions were also positive for sense RNA foci and/or poly(GA) inclusions supporting that the pTDP-43 pathology can be induced by (G4C2)_66_ transcript expression. Biochemical analysis of SDS-insoluble/ urea-soluble fraction showed that the pTDP-43 inclusions were predominantly in monomeric and dimeric forms indicating that the aggregation was at an early stage. Mild neurodegeneration was detected in cortex (17%), motor cortex (23%) and cerebellar Purkinje cells (11%) as quantified by NeuN-positive cells in these brain regions.

At 6 months of age, the AAV-G4C2-66 mice demonstrated increased anxiety, hyperactivity and reduced socialization behaviors compared to the AAV-G4C2-2 control mice. Significant impairment to motor coordination function as shown by Rota-Rod test was found in the AAV-G4C2-66 mice although no motor neuron loss was detected in spinal cord.

## Human BAC-Transgenic *C9orf72* Mouse Models

### *C9orf72* Exon 1–6 BAC (G4C2)_500_ SJL/B6 Mice do not Show Gross Behavioral and Neuropathological Abnormalities

Peters et al. (2015) used a bacterial artificial chromosome (BAC) DNA clone containing a partial human *C9orf72* gene region, including exons 1 to 6, a (G4C2)_500_ region and a 141 Kb 5′ upstream region, to generate a *C9orf72* HRE mouse model on SJL/B6 background. F6 progenies of the BAC-C9 mice stably carried a mix of (G4C2)_500_ and (G4C2)_300_ HRE containing genomic DNA in all tissues examined including brain and spinal cord. Based on the G4C2 repeat number, the mouse model is abbreviated as BAC-C9-(500/300) mouse model thereafter. The authors found that the levels of human *C9orf72* transgene RNA in the mouse model were similar to the endogenous mouse *C9orf72* RNA levels.

Both sense and antisense G4C2-repeat RNA foci were detected throughout the CNS including the spinal cord in the BAC-C9-(500/300) mice at 3, 10, and 24 months of age. Considerably less antisense RNA foci were detected compared to the numbers of sense RNA foci in the age-matched mice. Quantification of sense foci in the cortical internal pyramidal layer and spinal MNs showed that ∼50 and 80% nuclei contained RNA foci, respectively. A mild reduction of foci positive cells was detected in the 24-month-old mice compared to the 10-month-old BAC-C9-(500/300) mice.

The levels and distribution of soluble poly(GP) proteins were analyzed by the MSD immunoassay (Gendron et al., 2013). Highest levels were detected in cerebellum at 4 months (∼90 ng/mg protein). Relatively lower soluble poly(GP) levels were also detected in cortex, hippocampus and midbrain (∼50 ng/mg protein) at the same age. Lastly, the lowest poly(GP) levels were detected in hindbrain and spinal cord (∼30 ng/mg protein). Significant reduction of soluble poly(GP) levels were detected in 24-month-old mice compared to 4-month-old mice possibly due to an age-related decrease of poly(GP) solubility. Although not quantitative, poly(GP) inclusions were detected throughout the brain in cortex, striatum and cerebellum in 10-month-old mice and the inclusion number was apparently increased at 24 months as analyzed by IHC staining.

To investigate if the aged BAC-C9-500/300 mice exhibited any disease-related pathological and clinical phenotypes, the authors thoroughly examined any signs of neuronal cell death, neuroinflammation, TDP-43 related pathology, axonal and neuromuscular junction (NMJ) abnormalities, dendritic spine density changes at prefrontal cortex, electrophysiological deficits in neonatal cortical neurons, and transcriptome changes by genome RNA sequencing. However, no signs of any abnormality were detected.

Consistent with the negative pathological findings, no significant behavioral abnormalities were observed in the BAC-C9-(500/300) mouse model although the mice were examined for motor function abnormality throughout their lifespan and for socialization deficit at old age when compared to non-transgenic control mice.

### Full Length *C9orf72* BAC (G4C2)_100-1000_ C57BL/6J Mice Exhibit Alterations in Nucleolus and Immunomodulatory Genes

O’Rourke et al. (2015) used a BAC clone containing the human *C9orf72* locus, including all 11 exons, a (G4C2)_800_ region, 110 Kb 5′ upstream and 20 Kb 3′ downstream flanking regions, to generate a *C9orf72* HRE mouse model on C57BL/6J background. Two founder lines, F112 and F113, both with multiple G4C2-repeat species ranging from 100 to1000 repeats, were selected for subsequent analysis. A founder C57BL/6J line with 15 G4C2 repeats was used as control. Hence, the models here are abbreviated as BAC-C9-(100–1000) and BAC-C9-15 mouse models. Transgene expression levels were not quantified in the study. Because a ∼70–80% increase in total C9orf72 protein level was detected in these mice compared to that in non-transgenic control mice, transgene expression levels were estimated to be similar to that of endogenous mouse *C9orf72* RNA levels.

The F112 BAC-C9-(100–1000) mice expressed both sense and antisense G4C2-repeat RNA foci throughout the CNS including Purkinje cells in cerebellum, dentate gyrus (DG), frontal cortex, primary motor cortex and spinal cord MNs. About 40–80% of the cells examined were positive for sense and antisense RNA foci in different brain regions at 3 months of age. No significant changes in number and distribution of RNA foci were observed at age up to 8 months old.

Poly(GP) inclusions were rarely detected in the brain or the spinal cord at 6 months of age. At 20 months, poly(GP) cytoplasmic inclusions were detected in cortex, hippocampal DG and cerebellar granular layer of BAC-C9-(100–1000) mice. Using the MSD poly(GP) immunoassay (Gendron et al., 2013), soluble poly(GP) levels in cerebellum was twofold higher than in cortex (400 ng/mg protein vs. 200 ng/mg protein) and the levels were lower in hippocampus and spinal cord (i.e., 120 ng/mg protein and 100 ng/mg protein, respectively) at 6 months old.

Neuropathological examination of NMJs and femoral axon counts appeared normal. No changes were observed in protein aggregation (p62, TDP-43 and ubiquitin), gliosis, inflammation, and synaptic or neuronal loss in 18-month-old BAC-C9-(100–1000) mice. Consistently, the mice did not exhibit any abnormalities in motor function, anxiety, social behavior, and memory in 3- and 18-month-old mice.

Interestingly, the authors observed evidence supporting the presence of nucleolar stress in the BAC-C9-(100–1000) mice. A significant alteration of nucleolin protein distribution from nucleolus to a more disperse nucleus staining was observed in cerebellar Purkinje cells, and neurons of frontal and motor cortex. Transcriptome analysis for differential gene-expression showed significant downregulation of genes of immunomodulatory and extracellular matrix pathways in the BAC-C9-(100–1000) mice cortex compared to the non-transgenic controls. Genes involved in dendritic cell pathway, specifically regulating TH1 and TH2 cells development, were down-regulated in the BAC-C9-(100–1000) mice compared to non-transgenic control mice. Of note, the data was validated by a published dataset using induced pluripotent stem cells (iPSCs)-derived neurons from a patient with *C9orf72* mutation (Donnelly et al., 2013). Therefore, these molecular pathways might be potentially altered in C9FTD/ALS pathogenesis.

### *C9orf72* Exon 1–5 BAC (G4C2)_450_ Overexpression C57BL6/C3H Mice Display Robust C9ALS Neuropathology, Behavioral Deficits, and Mild Neurodegeneration

Jiang et al. (2016) generated a *C9orf72* HRE mouse model by expressing a BAC clone containing a partial human *C9orf72* gene with a (G4C2)_450_ repeat region in C57BL6/C3H mouse strain background. The BAC clone included a 140 Kb 5′ upstream region and a poly A sequence after exon 5 at 3′ end of the clone. One transgenic line with 110 repeats and three other lines with 450 repeats, namely BAC-C9-110, BAC-C9-450A, BAC-C9-450B, and BAC-C9-450C, were generated in this study. Human *C9orf72* RNA levels in the BAC-C9-450A mice were similar to the endogenous transcript levels in the cortex, while the BAC-C9-110, BAC-C9-450B, and BAC-C9-450C mice had 2.5, 3, and 4.5 folds higher transgene expression levels compared to the endogenous transcript levels in the same region, respectively.

Both sense and antisense RNA foci were detected throughout frontal cortex, hippocampus, cerebellum and spinal cord in 2-month-old BAC-C9-450B mice. About 50% more sense foci were in frontal cortex than in cerebellum. Although much less frequent, antisense RNA foci were detected in frontal cortex, hippocampus, cerebellum and spinal cord of BAC-C9-450B mice at 2 months. To understand how the transgene copy number affects RNA foci levels, the study found heterozygous BAC-C9-450C mice had about 52% cells with sense RNA foci whereas homozygous BAC-C9-450C mice had about 81% cells with sense foci in DG at 6 months of age. The BAC-C9-450C mice had about 29 and 42% cells with antisense foci in heterozygous and homozygous mice, respectively, at the same age. Interestingly, no RNA foci were detected in the BAC-C9-110 mice although the G4C2-repeat containing RNAs were three times higher in the BAC-C9-110 mice than in the BAC-C9-450B mice suggesting that the repeat length contributes to formation of RNA foci.

Analysis of C9RAN protein pathology in BAC-C9-450C heterozygous mice showed poly(GA), poly(GP), and poly(GR) cytoplasmic aggregates in 3-month-old mice in the retrosplenial cortex (RSCx). Age-dependent increases in the number and size of Poly(GA) aggregates in DG and RSCx were observed longitudinally from 6 to 22 months of age of the heterozygous BAC-C9-450C mice. Furthermore, the size of poly(GA) aggregates in DG and RSCx was at least doubled at 22 months compared to 6 months old mice. Age-dependent increase in the number of cells with poly(GA) aggregates was also observed in the BAC-C9-450C mice. For example, cells with poly(GA) aggregates were increased from about 2% to 28% in RSCx of heterozygous BAC-C9-450C mice during the 6–22 months age period. Poly(GA) and poly(GP) aggregates presented in about 30%, 10–20% and 5–10% of cells in RSCx, hippocampus and frontal cortex at 22 months, respectively. Very few poly(GA) and poly(GP) aggregates were detected in cerebellum and spinal cord. Poly(PR) and poly(PA) proteins were non-detectable by IHC. Using the MSD poly(GP) immunoassay, no soluble poly(GP) was detected in the BAC-C9-110 mice. The heterozygous BAC-C9-450C mice had about 45, 10, and 2 ng/mg of poly(GP) in the cortex, cerebellum and spinal cord at 6 months, respectively. Homozygous BAC-C9-450C mice had about 120, 30 and 20 ng/mg of poly(GP) in the cortex, cerebellum and spinal cord at the same age, respectively. Finally, reduced poly(GP) solubility during aging was detected in the heterozygous BAC-C9-450A and BAC-C9-450B mice as shown by an age-dependent reduction of soluble poly(GP) levels in cerebellum in from 2 to 16 months of age. The reduced solubility was expected to be consistent also for poly(GA) and poly(GR) proteins, as an age-dependent increase of aggregate size for poly(GP), poly(GA), and poly(GR) were observed in the RSCx of 3, 6, and 22 months old heterozygous BAC-C9-450C mice.

Although no TDP-43 protein inclusion was detected, increased pTDP-43 levels were detected in the SDS-insoluble/ sarkosyl-soluble fraction of cortex from 22-month-old heterozygous C9-450C mice. No neuromuscular unit dysfunction or MN loss was detected in the BAC-C9-450 mice.

About 10% neuronal loss was detected in DG and *Cornu Ammonis* 1 (CA1) of hippocampus at 12 months (but not at 4 months) in both heterozygous BAC-C9-450B and BAC-C9-450C mice to a similar extent. No neuronal loss was detected in age-matched BAC-C9-110 mice. Under behavioral tests, partial learning deficit was detected in both the BAC-C9-450B and BAC-C9-450C mice as revealed by the Barnes maze and the radial arm maze at 12 months of age. Increased anxiety was demonstrated by marble burying and elevated plus maze tests. However, there were no abnormalities in social interaction, recognition and communication abilities, and no deficits detected in behavioral tests for novel object recognition, fear conditioning and serial reversal learning.

### Full Length *C9orf72* BAC (G4C2)_500_ FVB/NJ Mice Model Robust C9ALS Neuropathology, Hind Limb Paralysis, and Motor Neuron Degeneration

Liu et al. (2016) generated a *C9orf72* HRE mouse model by integrating a BAC clone containing the human *C9orf72* gene (with all 11 exons) and a (G4C2)_850+_ repeat region into the genome of FVB/NJ mouse. The BAC clone also contained a 52 Kb 5′ upstream region and a 19 Kb 3′ downstream region of the gene. Four transgenic mouse founder lines namely BAC-C9-500/32, BAC-C9-500, BAC-C9-36/29 and BAC-C9-37 were used for subsequent analysis. BAC-C9-500/32 line had 2 transgene copies (i.e., 1 copy with 500 repeats and the other copy with 32 repeats); BAC-C9-500 line had a single copy of transgene with 500 repeats; BAC-C9-36/29 line had 4 transgene copies with either 36 or 29 repeats; and BAC-C9-37 mice had a single transgene with 37 repeats.

The BAC-C9-500 mice expressed similar levels of human *C9orf72* RNA compared to endogenous mouse *C9orf72* RNA levels in frontal cortex. The BAC-C9-500/32 mice, BAC-C9-36/29 mice and BAC-C9-37 mice expressed about 2, 2.5, and 0.5 times the levels of human *C9orf72* RNA levels compared to the endogenous mouse *C9orf72* RNA levels. Interestingly, quantification of antisense RNA levels in frontal cortex showed that the BAC-C9-500/32 mice, BAC-C9-500 mice and BAC-C9-36/29 mice had ∼40, 22, and 5 folds higher levels than that in the BAC-C9-37 mice by quantitative PCR analysis. A four to Sevenfold increase in antisense RNA levels was also found in spinal cord of the BAC-C9-500/32 and the BAC-C9-500 mice compared to that in the BAC-C9-37 mice.

Strikingly, about one-third of both female BAC-C9-500 and BAC-C9-500/32 mice developed an early-onset form of disease characterized by inactivity, weight loss, hind limb paralysis and death between 3 and 6 months of age. These mice were termed as “acute-onset rapidly progressive” by the authors. The high expression female BAC-C9-36/29 mice also showed a similar array of symptoms albeit with a later onset after 6 months of age (will be referred to as “acute-progress” from here onward). At 12 months, about 35, 35, and 28% declines in survival of the acute-progress female BAC-C9-500, BAC-C9-500/32 and BAC-C9-36/29 mice were observed, respectively. No such phenotypes were found in the BAC-C9-37 mice. In addition to the “acute-progress” mice, about 40–50% of both male and female BAC-C9-500 and BAC-C9-500/32 mice, and about 31% of female BAC-C9-36/29 mice, adopted a much slower progression of disease phenotypes. These mice also presented much milder symptoms compared to the acute-progress mice. Notably, reduced survival was still observed in the female slow-progress mice, but no premature death was observed in the slow-progress male mice.

Sense RNA foci were detected in almost 100% of NeuN-positive neuronal cells in cortex, hippocampus, cerebellum and spinal cord of 2-month-old BAC-C9-500 and BAC-C9-500/32 mice. In contrast, antisense RNA foci were mainly localized to brain regions with degeneration including layer V of the motor cortex, hippocampus, cerebellar Purkinje layer and interneurons in lateral and posterior horn of spinal cord, except for the lumbar spinal cord MNs. A sizeable proportion of glial cells were also positive for sense foci but not antisense foci. Interestingly, sense or antisense RNA foci were not detected in the acute-progress end-stage BAC-C9-36/29 mice arguing that neurotoxicity was not directly associated with the presence of RNA foci.

Poly(GA) aggregates were detected throughout the brain in acute-progress end-stage (∼5–7 months) symptomatic lines (BAC-C9-500, BAC-C9-500/32 and BAC-C9-36/29) using an aggregate-specific poly(GA) antibody, 27B11. Poly(GP) aggregates were also detected in the neocortex and thalamus of the BAC-C9-500 and BAC-C9-500/32 mice at the same age. Correlating the RAN protein pathology with disease severity, the authors found significantly more and larger poly(GA) aggregates in symptomatic acute-progress end-stage mice than in age-matched asymptomatic mice particularly in the RSCx.

Interestingly cytoplasmic and nuclear TDP-43 inclusions were found selectively in the degenerating neurons throughout the brain of end-stage acute-progress BAC-C9-500, BAC-C9-500/32 and BAC-C9-36/29 mice but not in the asymptomatic or non-transgenic mice.

Pathological characterization showed widespread neurode generation in the end-stage acute-progress BAC-C9-500, BAC-C9-500/32 and BAC-C9-36/29 mice. Severe neuronal cell reduction was found in neocortex (75% reduction in layer II/III motor cortex; 57% reduction in layer V motor cortex), CA and DG regions of hippocampus, Purkinje layer (65%) and molecular layer (28%) of cerebellum, and interneurons in the posterior horn of spinal cord (∼33%). In contrast, slow-progress end-stage mice (both male and female) had more local neuronal loss in neocortex and cerebellum (25% loss in Purkinje neurons) but not in hippocampus.

In the end-stage acute-progress female BAC-C9-500 and BAC-C9-500/32 mice (between 4 and 7 months), extensive motor neuron loss was detected in the lumbar spinal cord (∼50% loss) compared to age-match non-transgenic mice. Axonal degeneration, as shown by loss of axonal integrity and increased ratio of small/large axons, and denervation of NMJs in tibialis anterior (TA, 66%) and diaphragm (49%) muscles were also detected in these mice. Consistent with MN loss and axonal degeneration, the acute-progress BAC-C9-500 and BAC-C9-500/32 mice showed hind-limb gait abnormalities at 4 months. The disease further progressed to hind limb weakness, reduced grip strength and eventually hind limb paralysis at 7 months. Slow-progress C9-500 and C9-500/32 mice also showed MN loss in lumbar spinal cord (30%) and mild axonal and NMJ abnormalities at 12–17 months. The mice presented milder motor phenotypes such as kyphosis, reduced activity, hind limb clasping, and intermittent seizures at 12 months. Interestingly, the slow-progress mice, but not the acute-progress mice, also demonstrated increased anxiety after 12 months.

## The BAC-C9 Mouse Models: One Size Does Not Fit All

Multiple groups have generated BAC-C9 mouse models to study pathogenesis of C9FTD/ALS. This approach offers many advantages over the ectopic over-expression approach including the presence of endogenous transcription and translation regulatory sequences that are essential for spatiotemporal control of expression, as well as representation of the complete repertoire of potential splice variants encoded by the gene that might play a role in disease pathogenesis. There’s a possibility that repeat expansions may alter splicing enhancers and silencers in the residing introns leading to alterations in splice variants of the parent genes. Therefore, inclusion of complete sequence context of repeat expansions may be important to correctly model G4C2 ALS in mice. The 4 BAC-C9 DNA clones utilized for transgenic mice generation contained different G4C2-repeat lengths, and sizes of the 5′ and 3′ flanking genomic regions with either complete or partial *C9orf72* coding region. Although the BAC-C9 models were generated to study RNA and RAN-protein gain-of-toxicity, there is a slight possibility that potential regulatory elements in coding region and 3′ untranslated region may alter the stability or localization of the *C9orf72* transcripts.

### Effects of Repeat Length on RNA Foci and C9RAN Protein Pathologies

Repeat-length dependent expression of sense and antisense RNA foci was consistently observed in BAC-C9 mice from multiple groups. No RNA foci were detected in the BAC-C9-15 mice (O’Rourke et al., 2015), BAC-C9-110 mice (Jiang et al., 2016), BAC-C9-36/39 mice and BAC-C9-37 mice (Liu et al., 2016). However, expression of short repeat-length transcripts at high levels can overcome the size limitation (Chew et al., 2015). Sense RNA foci were detected in the cortex and other regions in all the BAC-C9 mice with longer repeat-lengths at 2–3 months of age (**Table 1**, E). Although antisense RNA foci were also detected in these mice, they were consistently less abundant compared to the sense foci in most models except for the BAC-C9-(100–1000) mice which displayed similar numbers of cells with antisense foci in different brain regions (O’Rourke et al., 2015) (**Table 1**, compare F to E). The distribution between sense and antisense RNA foci was similar in BAC-C9 models except for the acute-progress BAC-C9 mice in which the antisense foci were localized only to the degenerative brain regions with the exception of lower MNs in spinal cord (Liu et al., 2016) (**Table 1**, F). A recent report suggested that antisense RNA foci were more abundant than sense RNA foci and were correlated with TDP-43 pathology in cerebellar Purkinje neurons and MNs in the ventral horns of spinal cord in C9ALS patients (Cooper-Knock et al., 2015). Consistent with this report, Liu et al. (2016) reported that both of these regions were susceptible to degeneration in their mouse model. However, since differential antisense RNA foci distribution to the degenerative regions was not reported in other G4C2-repeat mouse models, the role of antisense RNA foci in neurotoxicity requires further validation.

Along with the RNA foci, BAC-C9 mice also exhibited C9RAN protein pathology, especially poly(GP) inclusions (**Table 1**, E and H). Poly(GP) inclusions were found in all long-repeat BAC-C9 mouse models in cortex, hippocampus and cerebellum. Poly(GA) inclusions were analyzed and shown in the BAC-C9-450C mice (Jiang et al., 2016) and all symptomatic BAC-C9 mice in Liu et al. (2016) study. Poly(GR) inclusions were only shown in the BAC-C9-450C mice in the RSCx region (Jiang et al., 2016) (**Table 1**, H). Two observations were consistent with the findings from human post-mortem brain tissue studies (Mackenzie et al., 2015; Davidson et al., 2016): (1) None of the BAC-C9 mice showed any overt correlations between C9RAN protein inclusions burden and degenerative brain regions or TDP-43 pathology; (2) C9RAN protein pathology was rarely found in spinal cord MNs. Interestingly, an age-dependent increase in number and size of poly(GP) inclusions were detected in the BAC-C9-(500/300) mice (Peters et al., 2015) and in the BAC-C9-(100–1000) mice (O’Rourke et al., 2015). Jiang et al. (2016) also showed that number of cells with poly(GA) inclusions increased ∼10 times and average inclusion size increased ∼2-fold over 16 months (**Table 1**, H). The age-dependent increase of C9RAN protein pathology in the BAC-C9 mouse models suggests that the rate of C9RAN protein production and propensity for aggregation exceeds the rate of protein turnover. Despite the lack of spatial correlations between degenerative regions and C9RAN protein pathology in humans and mice studies, how the rate of pathological accumulation correlates with disease progress is still an open question. The availability of the C9 mouse models has offered new opportunities to study longitudinal effect of C9RAN proteins on neurotoxicity.

### Variable Phenotypes and Penetrance in BAC-C9 Mice

Although transgenic expression of the G4C2 HRE containing human *C9orf72* RNA in the mouse brain was confirmed in all the BAC-C9 mice, only 2 out of the 4 models demonstrated any evidence of neurodegeneration and clinical symptoms resembling C9FTD/ALS. In Jiang et al. (2016) study, a mild 10% neuronal loss was detected in the DG and CA1 regions of the hippocampus in 12-month-old BAC-C9-450B and BAC-C9-450C mice (450 repeats, **Table 1**, J). As a result, a deficit in spatial learning ability and increased anxiety were observed. Interestingly, although BAC-C9-110 mice (110 repeats) had similar transgene expression levels to BAC-C9-450B mice (**Table 1**, D), no pathological or clinical phenotypes were observed indicating repeat length-dependence. In contrast, despite having same repeat lengths as the other symptomatic mice in the same study, the BAC-C9-450A mice did not show similar cognitive deficits likely due to much lower transgene expression levels than the BAC-C9-450B and BAC-C9-450C mice (**Table 1**, D). The transgene expression level-dependent disease pathogenesis is further supported by the asymptomatic BAC-C9-(500/300) mice and BAC-C9-(100–1000) mice in Peters et al. (2015) study and in O’Rourke et al. (2015) study, respectively. In both cases, the transgene transcript levels were similar to endogenous *C9orf72* ortholog and therefore similar to that in the BAC-C9-450A mice (**Table 1**, D). Hence, these pieces of evidence suggest that a critical concentration of the toxic RNA species achieved by higher transgene expression level is required for inducing neuronal death and disease-related symptoms in mice.

The Liu et al. (2016) study reported 3 symptomatic BAC-C9 mouse lines including BAC-C9-500, BAC-C9-500/32 and BAC-C9-36/29 mice. As described above, about one-third of the female mice developed an acute-progress phenotype (4–7 months). About 40% of male and female BAC-C9-500 and BAC-C9-500/32 mice, but only 31% of female BAC-C9-36/29 mice developed a slow-progress phenotype (from 12 to 17 months). These mice also demonstrated a repeat-length dependent effect on clinical symptoms. In the acute-progress mice, the shorter repeat-length BAC-C9-36/29 mice presented a milder phenotype with later onset motor deficits, weight loss and premature death compared to the longer repeat-length BAC-C9-500 and BAC-C9-500/32 mice (40 weeks vs. 20–40 weeks). Among the slow-progress mice, a much lower percentage of BAC-C9-36/29 mice were symptomatic compared to the longer repeat-length mice. However, although the repeat-lengths between BAC-C9-36/29 mice and BAC-C9-37 mice were similar, the former had sixfold higher transgene levels (i.e., C9-36/29 = 3-fold endogenous levels; C9-37 = 0.5-fold endogenous levels) (**Table 1**, D). Given that the BAC-C9-37 mice were asymptomatic compared to the age-matched non-transgenic controls, the difference in expression levels was likely to account for the phenotypic difference. Therefore, these data also support a repeat length- and transgene level-dependent effect on neurodegeneration and development of behavioral phenotypes.

Several studies on the BAC-C9 mouse models have reported longitudinal analysis of C9FTD/ALS specific features including G4C2-repeat containing RNA foci. No or modest changes were observed in the number and size of RNA foci with age in the BAC-C9 mice (O’Rourke et al., 2015; Peters et al., 2015) (**Table 1**, E). It suggests that cellular mechanisms exist that can continuously clear toxic RNA species by mechanisms such as nuclear export and degradation. The fact that there was no difference in frequency and distribution of RNA foci between symptomatic and asymptomatic BAC-C9 mice in Liu et al. (2016) study argues for existence of additional pathogenic mechanisms.

Interestingly, the phenotypic BAC-C9 mouse models in Jiang et al. (2016) and Liu et al. (2016) studies presented with relatively early C9RAN protein pathologies (3 and 5 months, respectively) versus the non-phenotypic BAC-C9 mice (**Table 1**, H). The non-phenotypic BAC-C9-(100–1000) mice in O’Rourke et al. (2015) study showed rare poly(GP) aggregates at 6 months whereas the other non-phenotypic BAC-C9-500/300 mice in Peters et al. (2015) study found widespread poly(GP) aggregates in 10-month-old mice (**Table 1**, H). Furthermore, severity of poly(GA) pathology, represented by inclusion-size and frequency, was strongly associated with clinical symptoms of the acute BAC-C9 mice when compared to age-matched asymptomatic mice from the same line (Liu et al., 2016). These results raise a possibility that early appearance of C9RAN protein inclusions may be a potential marker of disease severity. Given the feasibility and success of positron emission tomography (PET) scan probes to analyze amyloid and tau aggregate accumulation in Alzheimer’s disease patients (Herholz and Ebmeier, 2011; Saint-Aubert et al., 2017), development of PET scan probe for C9RAN aggregates might pave the road for effective diagnostics and use as biomarker for C9FTD/ALS patients. Indeed, C9RAN poly(GP) protein signature in CSF was recently proposed to be a potential biomarker for C9FTD/ALS patients (Gendron et al., 2017). Development of poly(GP) aggregate-specific PET imaging probes might allow for a more comprehensive and less invasive diagnosis of disease status in addition to the proposed biochemical analysis especially since multiple BAC-C9 mouse models have demonstrated an inverse relationship between soluble poly(GP) levels and poly(GP) pathology during aging (Peters et al., 2015; Jiang et al., 2016).

## AAV-G4C2-66 Mouse Model

The AAV-G4C2-66 mouse model is a G4C2-HRE DNA AAV transduction model. Based upon the fundamental molecular abnormality of the *C9orf72* mutation, the main purpose of the AAV-G4C2-66 mouse model is to examine the *in vivo* biological and molecular consequences of constitutive over-expression of the toxic (G4C2)_66_ containing RNA in CNS neurons starting at the neonatal stage. The mouse model recapitulated two c9FTD/ALS mutation-specific features in the neurons – repeat length-dependent accumulation of sense RNA foci composed mainly of G4C2-repeat RNA and inclusions composed of C9RAN proteins poly(GP), poly(GA), and poly(GR). Importantly, the AAV-G4C2-66 model also recapitulated a cardinal pathological hallmark of C9FTD/ALS – protein inclusions composed of endogenous pTDP-43. Because majority of cells with pTDP-43 inclusions were also positive for RNA foci or poly(GA) inclusions, the observation strongly supports that the pTDP-43 inclusions were induced by the presence of (G4C2)_66_ RNA, however, the mechanism remains unclear. Given that TDP-43 pathology is closely tied to neurodegeneration in FTD and ALS (Mackenzie et al., 2013, 2015), the model represents one of the few animal models that trigger aggregation of endogenous WT TDP-43 and will be useful for studying the mechanisms of endogenous TDP-43 aggregation. However, alternative splicing and geneexpression alterations due to TDP-43 dysfunction were not evaluated. The AAV-G4C2-66 mouse model also recapitulated the neurodegeneration and behavioral deficits of C9FTD/ALS patients to a certain extent. The expression and distribution of RNA foci, C9RAN and pTDP-43 protein inclusions partially overlapped with the brain regions exhibiting degenerative phenotypes (cortex, motor cortex and Purkinje cells). While some of these molecular characteristics may cause neurotoxicity, more detailed and systematic approaches are required to clarify exact pathways leading to the disruption of neuronal homeostasis and subsequent cell death in C9FTD/ALS. A particular caveat about this model is the matter of constitutive over-expression of G4C2 HRE driven by the CBA promoter. For example, the soluble poly(GP) protein levels in whole brain homogenate are ~50 times higher than that in post-mortem frontal cortex tissue from patients (i.e., 17,000 ng/mg protein in AAV-G4C2-66 mice vs. ~350 ng/mg protein in human frontal cortex) (O'Rourke et al., 2015). The spatiotemporal expression pattern, molecular pathogenesis and neurotoxicity resulting from disease-related life-long neuronal expression of G4C2 HRE RNA under *C9orf72* promoter is likely very different from that of the multiple copies of the constitutive, strong, and almost ubiquitous neuronal expression pattern in the AAV-G4C2-66 mice. The applicability of the model to delineate the actual disease pathogenesis will depend upon the successful validation of molecular abnormalities leading to neurotoxicity in other models including cortical neurons derived from patient iPSCs, other mouse models, and post-mortem tissues with various stages of disease severity.

## Difference Between the AAV-C9 and BAC-C9 Models

Human *C9orf72* HREs are a direct cause of FTD and ALS. All 5 mouse models reviewed above were generated to recapitulate the gain-of-toxicity effect of G4C2-repeat expression. Except for the AAV-G4C2-66 model, which adopts a somatic transgenesis approach, the other 4 models were generated by incorporation of human BAC-*C9orf72* DNA clone isolated from patient cells with G4C2-repeat expansion into the mouse germline using the BAC transgenenics approach (Heintz, 2000; Johnson and Wade-Martins, 2011). Production of the RNA transcripts containing sense G4C2- or antisense G2C4-repeat was controlled by the human *C9orf72* promoter and transcription regulatory elements in the gene. Due to the lack of information of developmental gene expression of *C9orf72* and its protein expression, it is unclear how the repeat containing transcripts are regulated during development. Nevertheless, the expression profile of the repeat transcripts is expected to closely match to that in human cells. On the other hand, G4C2-repeat RNA expression in the AAV-G4C2-66 mice is dictated by the neuronal tropism of the AAV9 capsid, efficient delivery of viral vector by CSF flow with P0 injection and strong constitutive activity of the CBA promoter (Aschauer et al., 2013; Ayers et al., 2015). There are a few major differences between the AAV-C9 and BAC-C9 models: (1) the expression levels of G4C2-repeat RNA; (2) the cell-type specific spatiotemporal expression profile; (3) Repeat lengths. As described above, the AAV-G4C2-66 model had much higher expression levels of shorter G4C2 repeats and a more ubiquitous expression scheme in the CNS neuronal cells whereas the BAC-C9 models express longer repeats in a *C9orf72* promoter dependent manner in numerous cell types during early as well as late developmental stages. Also, the AAV-G4C2 model did not show antisense RNA foci in any of the published studies using the related model (Chew et al., 2015; Zhang et al., 2016; Gendron et al., 2017). This implicates that transcription of antisense repeat DNA may be dependent upon either the activity *C9orf72* promoter or other enhancer elements within the gene locus, or longer repeat lengths affecting the secondary structure of the RNA, splice variants, etc.

*C9orf72* knockout mouse models from multiple recent studies have consistently implicated C9orf72 protein function in autophagy and immune regulation (Amick et al., 2016; Atanasio et al., 2016; O’Rourke et al., 2016; Rizzu et al., 2016; Ugolino et al., 2016; Yang et al., 2016; Ji et al., 2017). C9orf72 protein is highly expressed in myeloid cells and may participate in the regulation of microglial function and neuroinflammation (Atanasio et al., 2016; O’Rourke et al., 2016; Rizzu et al., 2016). Therefore, combined effects from *C9orf72* haploinsufficiency and repeat RNA expression may contribute to C9FTD/ALS pathogenesis in *C9orf72* HRE mutation carriers. It is noteworthy that the AAV-G4C2-66 model will not be able to model *C9orf72* haploinsufficiency in myeloid cells. On the contrary, although the BAC-C9 mice are likely expressing the HRE RNA and C9RAN proteins in myeloid cells, increased levels (rather than decreased) of C9orf72 protein are present in these mice due to overexpression. Therefore, second-generation C9FTD/ALS mice are required to model combined effects of *C9orf72* haploinsufficiency and RNA toxicity in the physiological relevant cell types. CRISPR/Cas9 based long HRE knock-in models may be generated to tackle this problem.

## Gender and Strain Effects

Amyotrophic lateral sclerosis is ∼1.6 times more prevalent in males (Williams et al., 2013). Since *C9orf72* HRE ALS mouse models may be used for preclinical studies in the future, it is essential to consider gender related differences in disease presentation in ALS mouse models for tailored therapeutics. Interestingly, both Chew et al. (2015) (AAV-G4C2) and Liu et al. (2016) (BAC-C9 with ∼500 repeats) studies have reported higher disease vulnerability to G4C2-repeat expression in female mice. In the AAV-G4C2-66 mice, there was a mild but significant reduction in body weight of the female mice compared to the female AAV-G4C2-2 control mice. The body weight reduction was not observed in the male counterparts. However, no sex difference was detected for neurodegeneration or behavioral phenotypes (Chew et al., 2015). In the Liu et al. (2016) study, about one-third of female BAC-C9-500 and BAC-C9-500/32 mice showed acute, progressive disease phenotypes related to C9FTD/ALS including gait abnormalities and weight loss at 4 months of age, hindlimb paralysis and early death beginning at 5 months of age. Furthermore, extensive loss of upper and lower MNs, cortical and hippocampal neurons were observed in 5–7 months old acutely progressive female mice (Liu et al., 2016). This is in contrast to the human disease. None of the other BAC-C9 models showed sex differences suggesting that genetic background of the mice may effect disease modeling. For example, both the BAC-C9-450B and BAC-C9-450C mice in Jiang et al. (2016) study had similar repeat numbers and higher transcript levels compared to the BAC-C9-500 mice in Liu et al. (2016) study (**Table 1**, D), however, the disease phenotypes in the former mice are much milder and no MN loss or motor function deficits were detected. The BAC-C9 mice in Liu et al. (2016) study and in Jiang study were in FVB/NJ strain and C57BL6/C3H strain, respectively. Notably, sex differences have indeed been reported in a mouse model of fragile X syndrome, a neurodevelopmental disorder, in FVB/NJ background (Reynolds et al., 2016). Nevertheless, it is important to delineate the effect of mouse strain on for better modeling of C9FTD/ALS.

## Neurotoxicity: RNA Foci Versus C9RAN Proteins

Relative contributions of RNA foci versus C9RAN proteins to neurotoxicity in C9FTD/ALS have been debated. Current transgenic mouse models provide little evidence for RNA foci-mediated toxicity. In O’Rourke et al. (2015) study, the authors failed to validate RBP sequestration and colocalization by sense- or antisense-RNA foci. While RNA foci localization overlapped with some of the neurodegenerative brain regions in the AAV-G4C2-66 mice and the BAC-C9-450C mice, RNA foci burden did not correlate with neuronal death in most of the brain regions under analysis. An exception to that observation is the acute-progress mice in Liu et al. (2016) study. The authors found that the frequency of antisense RNA foci was positively correlated with regions with neurodegeneration including layer V of frontal cortex, hippocampus, Purkinje cells and interneurons in lumbar spinal cord (Liu et al., 2016). As discussed earlier, all the phenotypic *C9orf72* HRE mice showed abundant C9RAN pathology at or before 6 months (**Table 1**, H). Interestingly, burden of poly(GA) inclusions correlated with onset of symptoms in the acute-progress mice in Liu et al. (2016) study and very few inclusions were detected in the CNS of age-matched asymptomatic mice.

Overall, although the creation of first-generation C9FTD/ALS mouse models mainly focused on creating valuable systems that recapitulate disease-related pathology and clinical features such as distribution and levels of RNA foci, C9RAN proteins, neurodegeneration, and behavioral deficits, more in-depth mechanistic interrogations at subcellular and molecular levels are expected in follow-up studies. Meanwhile, studies using Drosophila *melanogaster* models have provided more mechanistic insights on *C9orf72* HRE-induced toxicity. For example, a 160 G4C2 repeats Drosophila model with little C9RAN protein expression and ∼10 times more RNA foci than human neurons displayed minimal transcriptome changes and neuronal death (Tran et al., 2015). From the same study, over-expression of C9RAN protein mediated by a 36 G4C2 repeats-poly(A) construct displayed severe neurotoxicity in the other Drosophila line. Hence, the study teased out the neurotoxic effect of C9RAN protein from the RNA foci. In addition to this study, overwhelming evidence of C9RAN protein-mediated toxicity has tipped the balance in favor of DPRs vs. RNA foci (Mizielinska et al., 2014, 2017; Wen et al., 2014; Yang et al., 2015; Lee et al., 2016). However, levels of C9RAN proteins driven by strong promoters in these models may not recapitulate the low level sustained expression seen in the human disease.

## Nuclear-Cytoplasmic Transport

While the mouse models were characterized for *C9orf72* HRE induced C9FTD/ALS deficits, it will be valuable to explore the common downstream molecular and cellular pathways that link various etiologies to neurotoxicity during disease progression. Disruption of nucleocytoplasmic transport (NCT) has been reported and highlighted as a potential neurotoxic mechanism in C9FTD/ALS (Freibaum et al., 2015; Jovicic et al., 2015; van Blitterswijk and Rademakers, 2015; Zhang et al., 2015; Boeynaems et al., 2016; Prpar Mihevc et al., 2017). Of note, NCT abnormalities appear to be downstream of G4C2-repeat RNA and C9RAN proteins expression. RanGTPase-activating protein (RanGAP), a major component of NCT protein complex, was shown to bind G4C2 repeats RNA in a 30 G4C2-repeat Drosophila model and in iPSC-derived neurons from a C9ALS patient (Zhang et al., 2015). Importantly, consistent with compromised NCT, the ratio of nuclear to cytoplasmic TDP-43 protein levels was reduced – a pathological event leading to neuronal cytoplasmic TDP-43 inclusions and loss of nuclear TDP-43 in C9FTD/ALS patients. Notably, the BAC-C9-450C mice, which had no detectable motor unit or functional deficits, did not detect abnormalities in RANGAP cellular distribution or colocalization with RNA foci (Jiang et al., 2016). In contrast, the AAV-G4C2-66 mice that showed motor function deficits, showed RANGAP protein aggregates that predominantly colocalized with nuclear poly(GA) inclusions although NCT functional deficit was not confirmed in the study (Zhang et al., 2016). Furthermore, Poly(GA) inclusions in a pure C9RAN mouse model colocalized with Rangap1 and Pom121 (Zhang et al., 2016). Poly(PR) protein from antisense G2C4-repeat RNA was shown to compete with serine-arginine family of splicing factors (SR-proteins) for MTR10, a nuclear import receptor in the karyopherin protein family in a yeast genetic screen study (Jovicic et al., 2015). Lastly, long-term poly(GA) protein expression in neurons induced NCT deficits concomitant with TDP-43 mislocalization (Khosravi et al., 2017). Collectively, these findings suggest that C9RAN proteins may play a role in causing NCT dysfunction. Although these observations suggest that NCT deficits may appear early in C9ALS pathogenesis, the evidence available is correlative and circumstantial, and further validation in mouse models and human tissues is required to clarify the role of NCT dysfunction in C9FTD/ALS.

## Concluding Remarks

**FIGURE 1:**
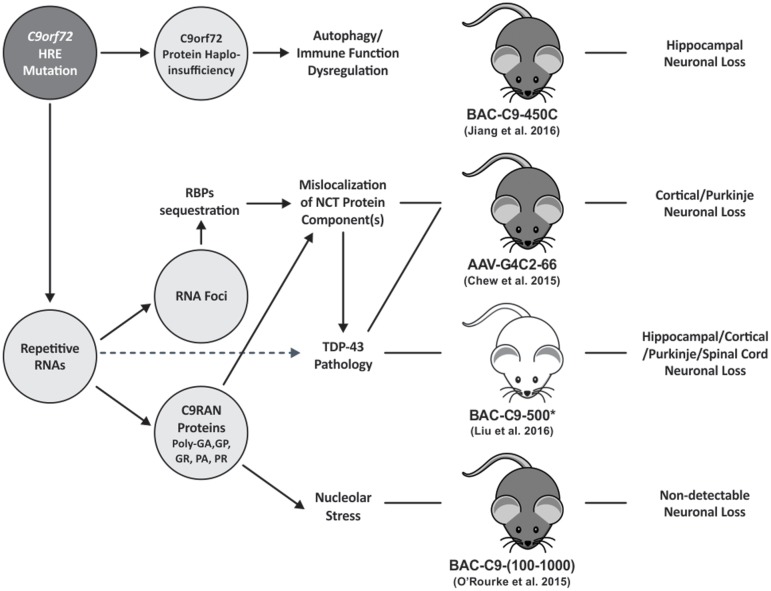
The schematic diagram summarizes the pathomechanisms of *C9orf72* HRE FTD/ALS. HRE containing RNAs are transcribed in C9FTD/ALS tissues. The repetitive RNA forms nuclear RNA foci or is exported to the cytoplasm where it undergoes RAN translation into poly-dipeptide-repeat C9RAN proteins. Like other microsatellite repeat expansion disorders, RNA foci induced by *C9orf72* HRE can sequester RBPs leading to their loss-of-function. G4C2-repeat RNA foci may alter the distribution and/or impair NCT function as shown in a mouse model (Zhang et al., 2016) and drosophila models (Freibaum et al., 2015; Zhang et al., 2015). Mislocalization of NCT components may also be caused by certain species of C9RAN proteins as shown in overexpression cell or animal models (Jovicic et al., 2015; Khosravi et al., 2017; Zhang et al., 2016). Notably, reduced nuclear-to-cytoplasm ratio of TDP-43 protein, a shuttling RBP, was detected in a of 30 G4C2 repeat overexpression drosophila model (Zhang et al., 2015) suggesting that TDP-43 pathology may be the result of impaired NCT function. However, additional mechanisms that link repetitive RNA expression with TDP-43 pathology are likely present. Elucidation of these molecular pathways is important as TDP-43 pathology burden is highly correlated with the severity of neurodegeneration (Neumann et al., 2006; Mackenzie et al., 2015). Furthermore, Arginine-rich C9RAN proteins [i.e., poly(GR), poly(PR)], were shown to induce nucleolar stress in cell and in drosophila models (Kwon et al., 2014; Wen et al., 2014; Lee et al., 2016). *C9orf72* HREs may cause loss of *C9orf72* gene-expression leading to haploinsufficiency. Multiple *C9orf72* knockout mouse models have consistently demonstrated the essential role of C9orf72 protein in autophagy and immune function regulation (Atanasio et al., 2016; Jiang et al., 2016; O’Rourke et al., 2016; Ugolino et al., 2016; Ji et al., 2017). But it is unclear how reduced C9orf72 protein function may contribute to *C9orf72* HRE FTD/ALS mechanism. Solid line arrows illustrate links supported by mechanistic data; whereas dotted line arrows illustrate links supported by correlation data. Pathological features reported in different *C9orf72* HRE mouse models are also illustrated. However, the pathomechanisms leading to neuronal loss in these models are still not clear. Mouse body color was selected based on strain information. Asterisk represents all acute-progress C9 mice in Liu et al. (2016) study. HRE, hexanucleotide repeat expansion; RBPs, RNA-binding proteins; NCT, nucleocytoplasmic transport.

The first generation G4C2-repeat expansion mouse models have shown tremendous promise on the feasibility of modeling C9FTD/ALS in mammals. Despite the variability in extents of neurodegeneration and clinical phenotypes, all the models displayed the C9FTD/ALS specific features of RNA foci and C9RAN protein inclusions, thus representing valuable tools to understand molecular pathogenesis of the disease. Differences in phenotypes of different models may stem from difference in genetic backgrounds of the mice. Backcrossing mice to congenic backgrounds will lead to better comparisons in the future. For the mouse models that showed neurodegeneration related phenotypes, it will be important to pinpoint the causes of neurotoxicity and carefully dissect disease related pathogenic mechanisms from phenotypes associated with artificial transgene expression levels, spatiotemporal patterns, and methodology. Models that directly test downstream events such as NCT, RNA splicing events, RBP dysfunction, and TDP-43 aggregation will give further credence to proposed mechanisms of C9FTD/ALS related neurotoxicity (**Figure 1**). Furthermore, validation of these findings in additional models such as iPSC-derived neurons and post-mortem tissues from C9FTD/ALS patients will not only bolster the findings of the mouse models but will also provide models for future diagnostic and therapeutic development.

## Author Contributions

CL and RB contributed equally to write and revise the manuscript.

## Conflict of Interest Statement

The authors declare that the research was conducted in the absence of any commercial or financial relationships that could be construed as a potential conflict of interest. The reviewer HO and handling Editor declared their shared affiliation, and the handling Editor states that the process nevertheless met the standards of a fair and objective review.
